# A Preliminary Fifth Year in the Medical Curriculum

**Published:** 1904-12

**Authors:** 


### A PRELIMINARY FIFTH YEAR IN THE MEDICAL CURRICULUM.

Dr. John Rogers, the secretary of the Cornell University Medical School, suggests a plan for securing a better preparation of students for the practice of medicine, which is worthy of consideration. From the statement that there is necessity of providing more time for both pupil and teacher—of demanding a better preparation for medical work—there can be no dissent.

With the conditions existing in medicine at the present time, it is reasonable and necessary to demand a longer period of preparation than is afforded by an ordinary high-school course, plus four years in the medical college. Medicine has made more progress in the direction of scientific exactitude in the last quarter of a century than in all its previous history, and needs for its intelligent study and comprehension a broader and more thorough training in the principles, facts and methods of the fundamental sciences than is afforded by the secondary schools.

We are in accord with Dr. Rogers' suggestion that an added preparation should be made in the premedical branches, for it is in these that the deficiencies of our present-day students and of the profession are most obvious and of most serious import. As to the proposal, however, that the instruction in the subjects should be given in the medical school rather than in the college or university proper, there is room for difference of opinion. It would seem that in such a step the medical school would be usurping the function which belongs properly and logically to the institution for general learning. The premedical subjects enumerated by our correspondent come logically within the domain of the college proper, and, with the possible exception of bacteriology and physiology, there are few, even of the smaller colleges, which are not well prepared to teach them—better equipped, indeed, both in faculty and in teaching appliances than the majority of medical colleges.

Medicine is applied science, its study and practice consisting in the application to the investigation and treatment of disease of the principles, facts and methods of certain fundamental sciences—among them physics, chemistry and the several biologic branches. Preparation for medicine, as for any other profession, naturally divides itself into a general education, whose chief purpose is the training of the faculties and the mastering of the fundamental subjects, and technical instruction in the application of these fundamental sciences to the specific problems of medicine.

General education is the province of the institution devoted to that purpose—the secondary schools and the college or university. The medical school should devote itself exclusively to the technical instruction, the applied science, and this it can only do to best advantage when its students have already mastered, in a broad and comprehensive manner, the principles and methods of the sciences to be applied. The curriculum of our better colleges includes those sciences fundamental to medicine. Is it a serious hardship to demand of every student intending to take up medicine that he spend one or two years in such a college? Surely a year in such an institution would be more economical and advantageous than the proposed extra year in the medical school. Such colleges are numerous in all portions of the United States, and, in the matter of tuition and cost of living, much less expensive than the majority of medical colleges.

If one year of such college work be demanded for admission to every medical school, a larger proportion of those who enter the college intending to take only this one year, will be inspired, and will find it possible to take one or two additional years—in many cases to secure the bachelor's degree and to pursue other studies which are nearly as essential to the adequate education of the successful physician as are the premedical branches above enumerated. Many medical students of the present time are unable to speak and write the English language as correctly as is befitting to the members of a learned profession; a majority have not had the training in mathematics which is essential to the thorough and intelligent comprehension of modern physics and chemistry; and a reading knowledge of German and of French is acknowledged to be of great advantage to the physician who is ambitious to be at the front. Our correspondent would hardly consider these subjects as coming within the province of the medical school, yet they are as important as some of the premedical sciences he mentions. It would be of far greater advantage, for example, for the student designing to enter the Cornell University Medical School to spend the suggested premedical year in the University at Ithaca than in the medical department in New York City.

The time has arrived when every medical college should demand at least one year of college work, in specified, essential branches, as a prerequisite for admission. It will, however, defeat the very purpose which is sought to be accomplished if, encroaching on the domain of the institution for general learning, the medical college attempts to give instruction in subjects entirely without its province and which it is wholly unfitted to undertake.

There is a fifth year, quite within the legitimate and logical sphere of the medical college, which, when the time is ripe, may with great advantage be required, and that is a practical clinical year, to be added to the further end of the present curriculum, and to consist of a hospital interneship. When the annual enrollment of medical students has been reduced to only such proportions as are actually needed to supply the demand; when the ratio of internes to patients in the larger hospitals has been doubled, as it must be to render adequate attention to their patients; and when the hospitals which are springing up so numerously in the smaller cities and towns throughout the country have created a greatly increased demand for internes, it will be easily possible to provide every medical student who has completed his fourth year in the medical college with a hospital service. Any technical difficulties —legal, educational or administrative—which may arise will find a ready solution. No such step should be undertaken, however, until an adequate premedical education, prescribed in the requirements for admission, has been fully and universally secured.

